# HMGA1 Induces Intestinal Polyposis in Transgenic Mice and Drives Tumor Progression and Stem Cell Properties in Colon Cancer Cells

**DOI:** 10.1371/journal.pone.0030034

**Published:** 2012-01-20

**Authors:** Amy Belton, Alexander Gabrovsky, Young Kyung Bae, Ray Reeves, Christine Iacobuzio-Donahue, David L. Huso, Linda M. S. Resar

**Affiliations:** 1 Hematology Division, Department of Medicine, The Johns Hopkins University School of Medicine, Baltimore, Maryland, United States of America; 2 Department of Medicine, The Johns Hopkins University School of Medicine, Baltimore, Maryland, United States of America; 3 Department of Pathology, Yeungnam University College of Medicine, Daegu, South Korea; 4 School of Molecular Biosciences, Washington State University, Pullman, Washington, United States of America; 5 Department of Pathology, The Johns Hopkins University School of Medicine, Baltimore, Maryland, United States of America; 6 Department of Oncology, The Johns Hopkins University School of Medicine, Baltimore, Maryland, United States of America; 7 Department of Molecular and Comparative Pathobiology, The Johns Hopkins University School of Medicine, Baltimore, Maryland, United States of America; 8 Department of Pediatrics, The Johns Hopkins University School of Medicine, Baltimore, Maryland, United States of America; Penn State Hershey Cancer Institute, United States of America

## Abstract

**Background:**

Although metastatic colon cancer is a leading cause of cancer death worldwide, the molecular mechanisms that enable colon cancer cells to metastasize remain unclear. Emerging evidence suggests that metastatic cells develop by usurping transcriptional networks from embryonic stem (ES) cells to facilitate an epithelial-mesenchymal transition (EMT), invasion, and metastatic progression. Previous studies identified HMGA1 as a key transcription factor enriched in ES cells, colon cancer, and other aggressive tumors, although its role in these settings is poorly understood.

**Methods/Principal Findings:**

To determine how HMGA1 functions in metastatic colon cancer, we manipulated *HMGA1* expression in transgenic mice and colon cancer cells. We discovered that HMGA1 drives proliferative changes, aberrant crypt formation, and intestinal polyposis in transgenic mice. In colon cancer cell lines from poorly differentiated, metastatic tumors, knock-down of HMGA1 blocks anchorage-independent cell growth, migration, invasion, xenograft tumorigenesis and three-dimensional colonosphere formation. Inhibiting *HMGA1* expression blocks tumorigenesis at limiting dilutions, consistent with depletion of tumor-initiator cells in the knock-down cells. Knock-down of HMGA1 also inhibits metastatic progression to the liver *in vivo*. In metastatic colon cancer cells, HMGA1 induces expression of *Twist1*, a gene involved in embryogenesis, EMT, and tumor progression, while HMGA1 represses *E-cadherin*, a gene that is down-regulated during EMT and metastatic progression. In addition, *HMGA1* is among the most enriched genes in colon cancer compared to normal mucosa.

**Conclusions:**

Our findings demonstrate for the first time that HMGA1 drives proliferative changes and polyp formation in the intestines of transgenic mice and induces metastatic progression and stem-like properties in colon cancer cells. These findings indicate that HMGA1 is a key regulator, both in metastatic progression and in the maintenance of a stem-like state. Our results also suggest that HMGA1 or downstream pathways could be rational therapeutic targets in metastatic, poorly differentiated colon cancer.

## Introduction

Despite recent progress in colorectal cancer screening and treatment, metastatic colorectal cancer remains a leading cause of cancer death worldwide [1]–[2]. The molecular mechanisms that enable cancer cells to metastasize are poorly understood, although emerging evidence indicates that transcriptional networks required for stem cell properties during embryogenesis are co-opted during metastatic progression [3]–[4]. Recent studies identified HMGA1 as a key transcription factor enriched in human embryonic stem (ES) cells [3], hematopoietic stem cells [5]–[8], refractory leukemia [6]–[7], [9] and high-grade/poorly differentiated cancers from the breast, brain, and bladder [3]. Moreover, tumors overexpressing *HMGA1* and eight other ES transcription factor genes had decreased survival, underscoring the importance of these genes in tumor progression [3]. More recently, we found that HMGA1 protein levels correlate with poor differentiation status and decreased survival in pancreatic cancer, further implicating HMGA1 in an undifferentiated, stem-like state and tumor progression [10]. The *HMGA1* gene encodes the HMGA1a and HMGA1b chromatin remodeling proteins, which function to modulate gene expression by altering chromatin structure and assembling transcription factor complexes at specific promoters [11]–[18]. Previous studies demonstrate that HMGA1 induces oncogenic properties in cultured cells [19]–[27] and causes aggressive tumors in transgenic mice [9], [28]–[32]. The precise molecular pathways regulated by HMGA1 in transformation, however, are only beginning to emerge and studies to elucidate HMGA1 transcriptional networks are likely to uncover fundamental pathways involved in tumor progression and development.

Here, we report for the first time that the HMGA1 drives proliferative changes and polyp formation in the intestines of transgenic mice and directs molecular pathways important in tumor progression and stem cell properties in human colon cancer cells. Taken together, these findings suggest that *HMGA1* promotes tumor progression in colon cancer by reprogramming colonic epithelium to a stem-like state.

## Materials and Methods

### Ethics Statement

All animal experiments were conducted in accordance with a protocol approved by the Johns Hopkins University Animal Care and Use Committee (protocol# MO08M263). All mice were housed in a sterile environment where they had free access to food and water as outlined in our institutional guidelines.

#### Quantitative reverse transcription-PCR (qRT-PCR) analysis

RNA was prepared as we described [9], [27]. The primers for *HMGA1* [28], *Twist*, *Vimentin*, *E-cadherin*, *FOXC2*, *Snail1*, *Slug*, *TCF-3*, *Twist1*, *Twist2*, *SFH1B*) were previously reported [4]. *GAPDH* primers are commercially available (Applied Biosystems). qRT-PCR reactions were done in triplicate and repeated at least once.

#### Western analysis

Western analyses were performed as we described [9], [19]–[20], [33] using a commercial HMGA1 antibody (Abcam, catalogue #AB4078) diluted 1∶1000 and β-actin (Cell Signaling, catalogue #4967) diluted 1∶1000 as an endogenous control.

#### Cell lines

HCT116 (CCL-247; American Type Culture Collection) and SW480 (CCL-228; American Type Culture Collection) were cultured as recommended. Transfected cells were selected in puromycin (3 ug/ml).

#### Growth Curves

Cellular growth rates were determined as we previously described [19]–[20], [26]–[29], [33].

#### Anchorage-independent cell growth in soft agar, migration, and invasion assays

These assays were performed as we described [19]–[20], [26]–[28], except that the migration assay was done with 60,000 cells/well and invasion assays were done with 20 ul of growth factor reduced matrigel.

#### Colonosphere assay

Colonosphere assays were performed as previously described [34].

#### *In vivo* metastasis assay

This assay was performed as previously described [35] with cells (10^5^) injected per mouse (NOD/Shi-*scid*/IL-2Rγ^null^; Jackson Laboratory).

#### Xenograft tumorigenicity

These studies were done in nude (nu−/−) mice as we described [19]–[20].

#### Vectors

The short hairpin RNA (shRNA) interference plasmid for *HMGA1* has been described [36]. The empty shRNA vector was used as a control.

#### Immunohistochemical analysis

Tissue sections (4 µm thick) from small and large intestines of transgenic and control mice were generated and deparaffinized in xylene and then hydrated in a graded alcohol series. Heat-induced epitope retrieval was performed in an autoclave for 10 minute in sodium citrate buffer (pH 6.0). Endogenous peroxidase activity was inactivated by incubation of the sections in 4% H_2_O_2_ for 5 minutes. After rinsing in phosphate-buffered saline, the sections were incubated with rabbit monoclonal anti-Ki-67 antibody (clone 30-9, Ventana, Tucson, AZ, catalogue #790-4286) for 1 hour at room temperature. Positive staining was visualized with the DAKO EnVision Plus-HRP detection kit (DAKO, Carpinteria, CA) according to the manufacturer's instructions. Ki-67 positive cells were counted in 5 randomly selected fields from comparable areas in the small and large intestine from transgenic and control mice at 20× magnification. A total of 100 crypts for each mouse were counted from stained sections from transgenic and control mice (2 mice/group).

#### Cell morphology and size determinations

Cells were plated and allowed to grow to 80% confluency. Photographs for morphologic assessment and diameter measurements were obtained using the Zeiss Inverted M-scope with the Olympus DP72 Camera and Software (JHMI Confocal Facility). At least five random fields were photographed for each cell line with or without HMGA1 knock-down. The greatest diameter (µm) was measured for 700–1000 cells from each group. The greatest diameters were represented as the mean ± the standard deviation; significance was determined using the student's t-test.

## Results

### *HMGA1a* transgenics develop polyps and expansion in the stem cell compartment

**Figure 1:**
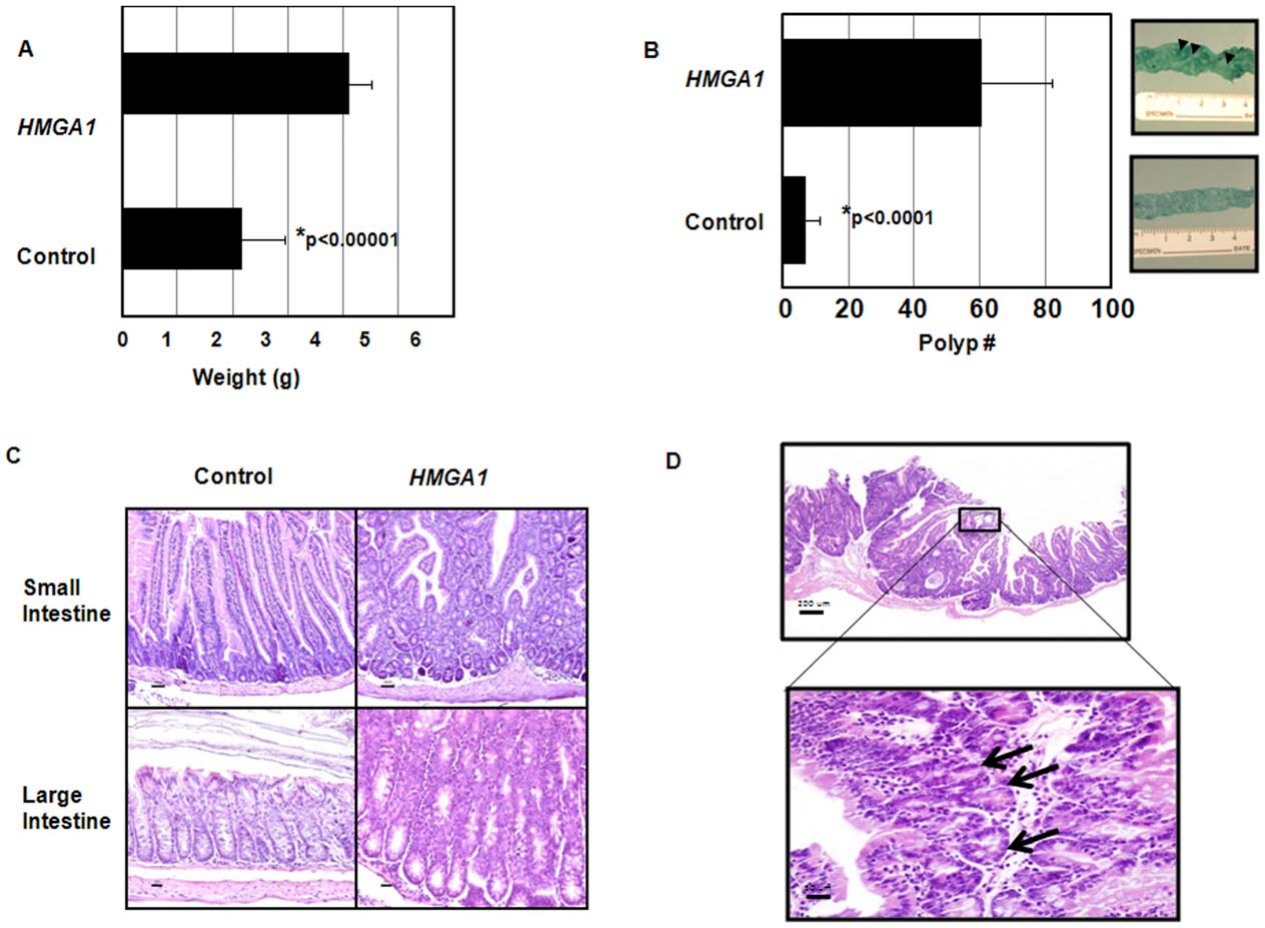
*HMGA1* causes proliferative changes, ectopic crypt formation, and polyps in transgenic mice. (A) The *HMGA1* transgenic intestines are hyperemic with increased weight compared to controls (n = 7 in each group; p = 0.000000138 by student's t-test.) (B) Polyp number in the transgenics and controls (n = 7 in each group; p = 0.000426 by student's t-test). The intestinal spreads (right panels) are stained with 1.5% methylene blue to accentuate polyps for counting. (C) Hematoxylin and eosin staining of the small and large intestine shows marked proliferative changes in the transgenics (bar = 50 µm). (D) Expansion of the proliferative zone resulting in a diffusely hyperplastic appearance of the mucosa and cystically dilated crypts in the transgenics. The higher power view of the small intestinal mucosa shows formation of ectopic crypt foci (arrows).

**Figure 2:**
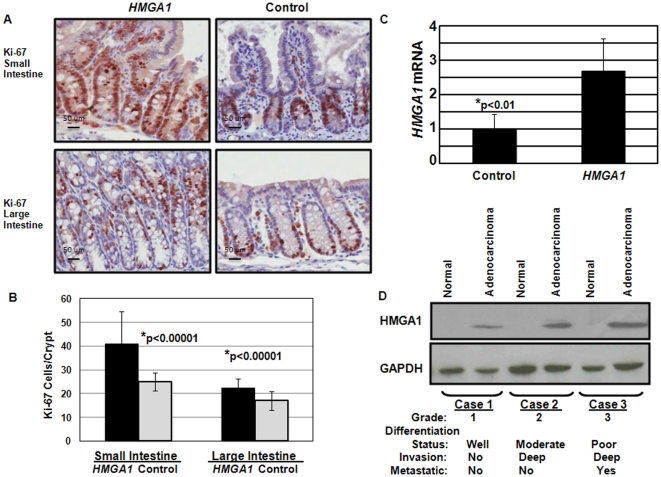
*HMGA1* drives proliferation in intestinal mucosa and is overexpressed in colon cancer. (A) Ki-67 staining of small and large intestines from *HMGA1* transgenics (bottom panels) and control mice (top panels) show a significant increase in Ki-67 immunoreactivity in the *HMGA1* transgenics (20× magnification). (B) Ki-67 positive cells in *HMGA1* transgenic and control mice were enumerated in crypts of the small and large intestine at 20× magnification. The mean ± the standard deviations of Ki-67 positive cells per crypt for 100 crypts for each pair of mice at each location are shown. Highly significant differences were demonstrated between transgenics and controls in both the small and large intestines (p<0.00001, student's t-test) (C) *HMGA1* expression was assessed by qRT-PCR and increased in the primary, high grade (grade 3–4) colon adenocarcinomas compared to adjacent, normal colonic tissue. *β-actin* was used to control for loading and the normal tissue was arbritrarily assigned a value of 1.0. (D) Western blot analysis for HMGA1 and control protein (GAPDH) was performed on 3 tumors with sufficient sample and showed increasing HMGA1 protein with decreasing differentiation and increasing invasiveness or frank metastasis. The grade, differentiation status, invasiveness, and presence of metastatic disease are indicated.

To define the role of HMGA1 in tumorigenesis, we engineered transgenic mice with the murine *hmga1a* transgene driven by the H2K promoter and immunoglobulin mu enhancer [9], [27]–[29]. As previously reported, all mice succumb to aggressive lymphoid malignancy [9] and the females develop uterine sarcomas [28]–[29]. To determine if HMGA1 promotes neoplastic transformation in intestinal epithelium, we investigated the intestines of these mice. The transgene is expressed in the small and large intestines by 4 to 7-fold above that found in controls (Fig. S1). At necropsy, the intestines are hyperemic with a thickened mucosa and increased weight compared to controls (Fig. 1A–D). Histologically, both large and small intestines exhibit marked proliferative changes, ectopic crypt formation, and polyp formation (Fig. 1C–D). There was a significant increase in Ki-67 in both the small and large intestine, a marker of proliferation (Fig. 2A–B). The increase in crypt number suggests that these mice could have expansion of the intestinal stem cell compartment [37].

### *HMGA1* is enriched in human colon carcinomas

High levels of HMGA1 protein or mRNA were reported in colon cancer cell lines or primary tumors in a small pilot study [38]. To determine if *HMGA1* is overexpressed in larger studies of primary colon cancers, we queried gene expression profile analysis of primary colon cancers from six independent studies through the Oncomine™ public database (Compendia Bioscience, Ann Arbor, MI). We found that *HMGA1* is highly enriched in colon adenocarcinoma compared to normal tissue in 5/5 prior studies. In all of these studies, HMGA1 was among the top 10% of enriched genes, and in 4 studies, it was among the top 1–3% of enriched genes. In a pilot of 10 primary, high-grade (grade III–IV) colon cancers (a generous gift from Bert Vogelstein), we found that HMGA1 expression was increased in most colon cancer samples (8/11) compared to adjacent normal tissue from the same patient. Moreover, the mean HMGA1 expression from all tumors was increased by 3-fold compared to the adjacent control tissue (Fig. 2C). We also assessed protein expression in a subset of primary colon tumors with sufficient material and found the highest levels of HMGA1 protein in the high grade, metastatic tumors with undetectable levels in the adjacent normal tissue (Fig. 2D).

### HMGA1 is required for anchorage-independent cell growth, migration, invasion, and tumorigenesis in colon cancer cells

**Figure 3:**
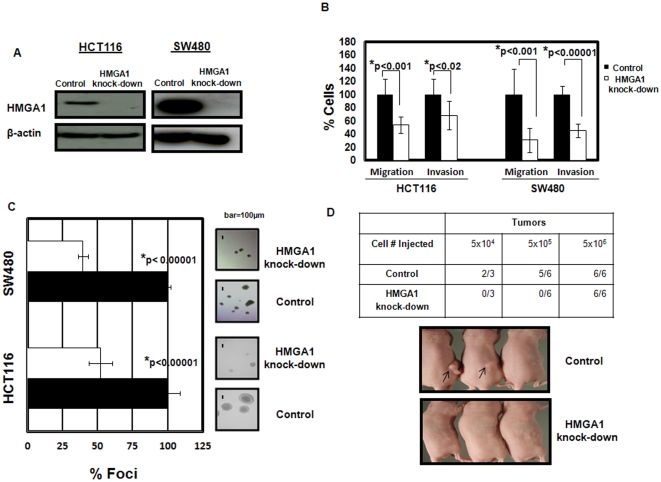
HMGA1 is required for migration, invasion, anchorage-independent cell growth, and limiting dilution tumorigenicity. (A) Transfection with an shRNA knockdown vector for *HMGA1* in highly aggressive, poorly differentiated human colon cancer cells (HCT116 and SW480) results in a significant decrease in HMGA1 protein. (B) Migration and invasion were assessed in control (dark bars) and HMGA1 knock-down (open bars) colon cancer cells (HCT116 and SW480). The graph shows the mean ± the standard deviation from 2 experiments done in triplicate. (p-values determined by student's t-tests are shown). (C) Anchorage-independent cell growth in soft agar in control (dark bars) and HMGA1 knock-down (open bars) colon cancer cells (HCT116 and SW480). All foci >50 µm were counted. The bar graph shows the mean ± the standard deviation from two experiments done in triplicate. (p-values determined by student's t-tests are shown). (D) The table shows that knock-down of HMGA1 blocks tumor formation at limiting dilutions. The photograph shows representative nude mice 4 weeks following injection with control or HMGA1 knock-down in HCT116 cells (10^5^/injection). No tumors formed in the HMGA1 knock-down cells.

To investigate the functional role of HMGA1 in colon cancer, we inhibited *HMGA1* expression in two colon cancer cell lines derived from poorly differentiated (grade 4), metastatic colon cancers (HCT116 and SW480) using an shRNA approach. Transfection of cells with an *HMGA1* shRNA vector resulted in a significant, stable decrease in HMGA1 protein (Fig. 3A) in the shRNA cells compared to cells transfected with the control vector. We found that knock-down of HMGA1 blocked anchorage-independent cell growth or foci formation in soft agar, migration, and invasion in both HCT116 and SW480 cells (Fig. 3B–C). Moreover, no tumors formed in nude mice after injection of HCT116 cells with HMGA1 knock-down (10^5^ or 10^4^ cells), while tumors formed from the cells transfected with control vectors (Fig. 3D). There was no significant difference in growth rates in the cell lines with or without HMGA1 knock-down *in vitro* (Fig. S2), indicating that the *HMGA1* shRNA was not toxic to the colon cancer cells. These results demonstrate that HMGA1 drives cellular properties required for both tumor initiation (anchorage-independent cell growth, tumorigenesis) and tumor progression (migration, invasion).

### HMGA1-dependent stem cell properties in colon cancer cells

**Figure 4:**
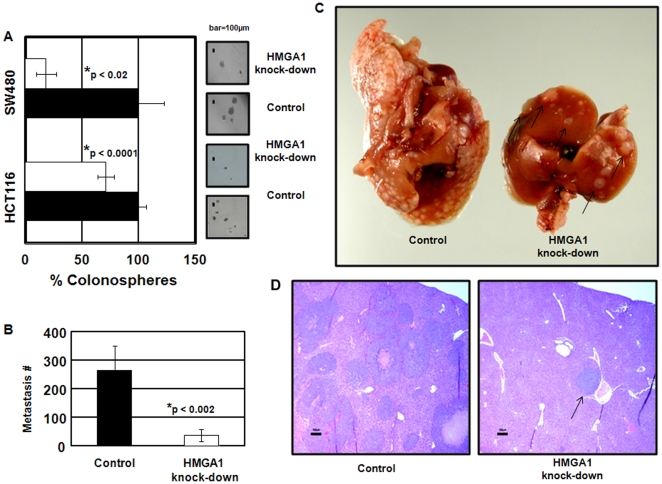
HMGA1 is required for colonsphere formation and metastatic progression *in vivo*. (A) Three-dimensional colonosphere formation in control (dark bars) and HMGA1 knock-down (open bars) colon cancer cells (HCT116 and SW480). Spheres were counted after 10 days. The graph shows the mean ± the standard deviation from 3 experiments done in triplicate. (p-values determined by student's t-tests are shown). (B) Metastatic foci in the liver were counted 5 weeks after injection of control (dark bars) or HMGA1 knock-down (open bars) HCT116 cells (1×10^5^) into the spleens of nude mice. The bar graph shows the mean ± the standard deviation of metastatic foci from two experiments done in triplicate. The mice injected with control cells (black bar) were compared to mice injected with HMGA1 knock-down cells (open bars; p-values determined by student's t-tests are shown). (C) Gross photographs of metastatic foci to the liver following injection of HCT116 cells into the spleens of jude mice. The left liver is taken from a representative mouse injected with control cells; the right liver is taken from a representative mouse injected with HMGA1 knock-down cells. Photographs depict representative livers from mice receiving control or *HMGA1* knock-down cells. (D) Histologic examination of the livers confirmed that the metastatic foci are significantly increased from the spleens injected with control cells compared to HMGA1 knock-down cells (Bar = 100 µm).

Because *HMGA1* is enriched in stem cells [5]–[8] and causes changes in the mouse intestine that could be consistent with expansion in the intestinal stem cell compartment, we sought to determine if *HMGA1* is involved in stem cell properties in colon cancer cells. To this end, we assessed three-dimensional colonosphere formation in the colon cancer cell lines with or without knock-down of HMGA1. We discovered a significant decrease in the number of colonospheres in both the HCT116 and SW460 cells with HMGA1 knock-down compared to controls (Fig. 4A). These findings indicate that HMGA1 is necessary for this stem cell phenotype (three-dimensional growth) in colon cancer cells. In limiting dilution tumorigenicity experiments, knock-down of HMGA1 blocked tumor formation when 104 or 105 cells were injected, whereas tumors formed in the control cells. Tumors formed in both control and knock-down groups if 106 cells were injected (Fig.3D). These results indicate that knock-down of HMGA1 depletes the tumor-initiator cells.

### HMGA1 is required for metastatic progression in colon cancer

To determine if HMGA1 is required for tumor progression, we used a preclinical model for metastatic progression in colon cancer [35]. HCT116 colon cancer cells were injected directly into the spleens of immunodeficient mice and liver metastases were assessed after 5 weeks. Strikingly, we found a marked decrease in metastatic foci in the livers from mice injected with HMGA1 knock-down cells (Fig. 4B–C). These results underscore the critical role of HMGA1 in metastatic progression in this model.

### HMGA1 induces expression of the EMT genes *Twist1* and *Vimentin*

**Figure 5:**
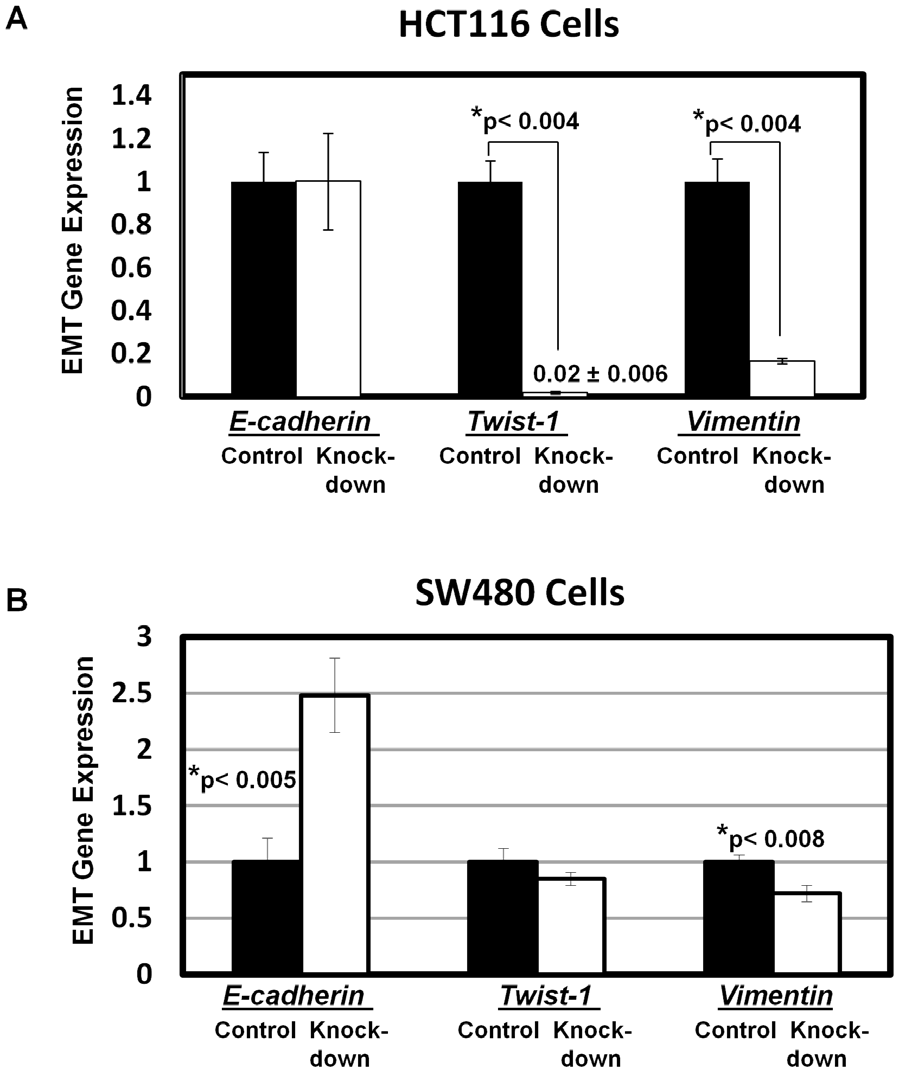
HMGA1 dysregulates genes involved in EMT in metastatic colon cancer cells. (A) In HCT116 cells, *Twist1*, and *Vimentin* mRNA expression were repressed by qRT-PCR in the HMGA1 knock-down cells compared to control cells. *E-cadherin* and other EMT genes were not affected in these cells. (p-values determined by the student's t-tests are shown). (B) In SW480 cells, *Vimentin* is repressed and *E-cadherin* is induced in the cells with HMGA1 knock-down. (p-values determined by the student's t-tests are shown).

Because recent studies link EMT to epithelial stem cell properties [3]–[4], we sought to determine if HMGA1 regulates EMT genes in colon cancer. To this end, we assessed expression levels of 11 genes previously shown to play an important role in EMT and cancer initiator cells [4]. In the HCT116 cells, we found that both *Twist1* and *Vimentin* were significantly repressed in cells with HMGA1 knock-down (Fig. 5A), indicating that HMGA1 induces their expression. In SW480 cells, *E-cadherin* is up-regulated in the knock-down cells, suggesting that HMGA1 normally represses its expression (Fig. 5B). *Twist1* was also significantly repressed in the SW480 knock-down cells, although less than what we observed in the HCT116 cells. Taken together, our studies suggest that HMGA1 promotes tumor progression through transcriptional networks that facilitate metastatic progression and a stem-like state, at least in part, by inducing the *Twist1* and *Vimentin*, while repressing *E-cadherin*. Of note, there were no changes in cell morphology or size in the knock-down cells (Fig. S3), suggesting that down-regulation of HMGA1 in these cells is not sufficient to induce morphologic changes consistent with a reversion to a more epithelial state when grown as a monolayer. As noted previously, there were significant alterations in growth characteristics when grown in an anchorage-independent fashion or after implantation into mice. The variation in EMT genes modulated by HMGA1 in these two different cell lines likely reflects the differing milieu and reprogramming potential in the cancer cells based on underlying genetic and epigenetic changes.

## Discussion

Metastatic colon cancer is highly lethal and the incidence is rising, particularly in younger individuals [1]–[2]. Current therapies are limited by the emergence of metastatic cancer cells that are resistant to treatment. Recent evidence suggests that these refractory cells develop because they co-opt the cellular networks involved in embryonic development and become capable of metastasizing and evading therapy [3]–[4]. The *HMGA1* oncogene was recently identified as a key transcription factor enriched in ES cells and high grade tumors with poor outcomes [3], although its function in these settings has remained elusive. This gene is a member of the *HMGA* gene family, that also includes *HMGA1* [11]–[18] and *HMGA2* [31], [39]–[40]. All HMGA proteins are small, low molecular weight (thus high mobility group proteins) with an AT-hook DNA binding domain that mediates binding to AT-rich regions in the minor groove of chromatin. HMGA1 and other AT-hook proteins are thought to function by modulating chromatin structure and gene expression [15]–[18], [41]–[42]. Here, we show for the first time that *HMGA1* induces proliferative changes and polyp formation in the intestines of transgenic mice. Moreover, *HMGA1* is required for limiting dilution tumorigenesis *in vivo* along with 3-dimensional colonosphere formation *in vitro*, both of which are phenotypes characteristic of epithelial stem cells. Furthermore, inhibiting *HMGA1* expression blocks not only transformation properties involved in tumor initiation (anchorage-independent cell growth, tumorigenicity), but also cellular characteristics that promote metastatic progression (invasion and migration). We also discovered that *HMGA1* is required for metastatic progression to the liver *in vivo*. Notably, several recent studies have also shown that *HMGA1* is enriched in normal stem cells, including embryonic and hematopoietic stem cells [3]–[8], in addition to poorly differentiated, or refractory stem-like cancers [8]–[32], suggesting that HMGA1 helps to drive a stem-like state, both in normal development and cancer. *HMGA1* is also highly expressed during embryogenesis, with low or undetectable expression in most differentiated, adult tissues [43].

To determine how HMGA1 orchestrates tumor progression and stem cell phenotypes, we investigated expression of genes that have been previously shown to be important in EMT, development, and tumor initiator cells [4]. In HCT116 cells, we found that HMGA1 up-regulates expression of both *Twist1* and *Vimentin*. In SW480 cells, HMGA1 also up-regulates *Twist1*, while it represses *E-cadherin*. *E-cadherin* did not change in the HCT116 colon cancer cells with knock-down of *HMGA1*, which could reflect stable silencing of *E-cadherin* in these mesenchymal, highly metastatic colon cancer cell lines. The transcriptional networks regulated by HMGA1 likely depends upon the cellular milieu and underlying genetic and epigenetic characteristics.

In summary, our studies provide the first evidence linking HMGA1 to cellular properties and transcriptional networks important in stem cells, EMT, and metastatic progression in colon cancer. Although further work is needed, these results underscore the role of HMGA1 as a key regulator in tumor progression and a stem-like state in colon cancer and suggest that targeting HMGA1 pathways could be beneficial in therapy for colon cancer. Because *HMGA1* is enriched in embryonic stem cells, tissue-specific stem cells, and virtually all aggressive tumors studied to date, our findings are likely to relevant not only to diverse human cancers, but also to normal development.

## Supporting Information

Figure S1 ***HMGA1*** **transgene expression throughout the small and large intestines in the transgenic (TG) mice compared to wildtype (WT) controls.** qRT-PCR for *HMGA1* and the control gene, *GAPDH*, was performed from tissue obtained throughout the small intestine (duodenum, jejunum, and ileum) and large intestine (ascending colon, designated A colon, and descending colon, designated D colon) from the TG and WT mice. *HMGA1* expression in the TG intestines is increased by 4 to 8-fold above that observed in the WT mice, which was arbitrarily assigned a value of 1. All qRT-PCR reactions were done in triplicate and repeated at least once.
(TIF)

Figure S2 **Proliferation rates in HCT116 or SW480 colon cancer cells with HMGA1 knock-down or control vector.** (A) Growth rates of the HCT116 cells transfected with the *HMGA1* shRNA vector to knock-down HMGA1 (red squares) or control vector (blue circles) show a similar proliferation rate. Standard deviations are ≤10% and therefore not visible. (B) Growth rates of the SW480 cells transfected with the control vector (blue circles) or *HMGA1* shRNA vector (red squares) show a similar proliferation rate. Standard deviations are ≤10% and therefore not visible.
(TIF)

Figure S3 **HMGA1 knock-down results in alterations in EMT genes without changes in cell morphology.** (A) Knock-down of HMGA1 in HCT116 cells does not result in changes in cell morphology or diameter compared to controls. (B) Knock-down of HMGA1 in SW480 cells does not result in changes in cell morphology or diameter compared to controls. Photographs were taken of live cells using a 10× objective; scale bar, 100 µm.
(PPTX)

## References

[1] Jemal A, Siegel R, Ward E, Y Hao Y, Xu J (2010). Cancer statistics.. CA Cancer J Clin.

[2] Fearon ER (2011). Molecular genetics of colorectal cancer.. Annu Rev Pathol Mech Dis.

[3] Ben-Porath I, Thomson MW, Carey VJ, Ge R, Bell GW (2008). An embryonic stem cell-like signature in poorly differentiated aggressive tumors.. Nat Genetics.

[4] Mani SA, Guo W, Liao MJ, Ng E, Ayyanan A (2008). The epithelial-mesenchymal transition generates cells with properties of stem cells.. Cell.

[5] Zhou G, Chen J, Lee S, Clark T, Rowley JD (2001). The pattern of gene expression in human CD34^+^ stem/progenitor cells.. Proc Natl Acad Sci USA.

[6] Karp JE, Smith BD, Resar LMS, Greer JM, Blackford AL (2011). Phase I and pharmacokinetic study of “hybrid” (bolus-infusion) flavopiridol administered followed in time sequence by cytosine arabinoside and mitoxantrone for adults with relapsed and refractory acute leukemias.. Blood.

[7] Nelson DM, Joseph B, Hillion J, Segal J, Karp JE (2011). Flavopiridol induces *BCL2* expression and represses oncogenic transcription factors in leukemic blasts from adults with refractory acute myeloid leukemia.. Leuk Lymphoma.

[8] Chou BK, Mali P, Huang X, Ye Z, Dowey SN (2011). Unique epigenetic signature of human blood cells permits efficient iPS cell derivation by a non-integrating plasmid.. Cell Research.

[9] Xu Y, Felder TS, Bhattacharya R, Tesfaye A, Fuchs E (2004). The HMG-I oncogene causes highly penetrant, metastatic lymphoid malignancy in transgenic mice and is overexpressed in human lymphoid malignancy.. Cancer Res.

[10] Hristov A, Cope L, Di Cello F, Delos Reyes M, Singh M (2010). HMGA1 correlates with advanced tumor grade and decreased survival in pancreatic ductal adenocarcinoma.. Mod Pathol.

[11] Johnson KR, Cook SA, Davisson MT (1992). Chromosomal localization of the murine gene and two related sequences encoding high-mobility-group I and Y proteins.. Genomics.

[12] Johnson KR, Lehn DA, Elton TS, Barr PJ, Reeves R (1988). Complete murine cDNA sequence, genomic structure, and tissue expression of the high mobility group protein HMG-I(Y).. J Biol Chem.

[13] Friedmann M, Holth LT, Zoghbi HY, Reeves R (1993). Organization, inducible-expression and chromosome localization of the human HMG-I(Y) nonhistone protein gene.. Nucleic Acids Res.

[14] Pedulla ML, Treff NR, Resar LMS, Reeves R (2001). Cloning and comparative sequence analysis of the murine *Hmgiy* (*Hmga1*) gene.. Gene.

[15] Hock R, Furusawa T, Ueda T, Bustin M (2007). HMG chromosomal proteins in development and disease.. Trends Cell Biol.

[16] Reeves R (2001). Molecular biology of HMGA proteins: hubs of nuclear function.. Gene.

[17] Fusco A, Fedele M (2007). Roles of HMGA proteins in cancer.. Nat Rev Cancer.

[18] Resar LMS (2010). The *High Mobility Group A1* Gene: Transforming inflammatory signals into cancer?.

[19] Wood LJ, Mukherjee M, Dolde CE, Xu Y, Maher JF (2000). HMG-I/Y: A new c-Myc target gene and potential human oncogene.. Mol Cell Biol.

[20] Wood LJ, Maher J, Bunton TE, Resar LMS (2000). The oncogenic properties of the HMG-I gene family.. Cancer Res.

[21] Reeves R, Edberg D, Ying L (2001). Architectural transcription factor HMGI(Y) promotes tumor progression and mesenchymal transition of human epithelial cells.. Mol Cell Biol.

[22] Dolde CE, Mukherjee M, Cho C, Resar LMS (2002). The role of HMG-A1/Y in the human breast cancer.. Breast Cancer Res Treat.

[23] Takaha N, Resar LMS, Vindivich D, Coffey DS (2004). High mobility protein HMGI(Y) enhances tumor cell growth, invasion, and matrix metalloproteinase-2 expression in prostate cancer cells.. The Prostate.

[24] Hommura F, Katabami M, Leaner VD, Donninger H, Felder Sumter T (2004). HMG-I/Y is a cJun/AP-1 responsive gene and is necessary for cJun induced anchorage-independent growth.. Mol Cancer Res.

[25] Dhar A, Hu J, Reeves R, Resar LMS, Colburn N (2004). Dominant negative c-Jun (TAM67) target genes: HMGA1 is required for tumor promoter-induced transformation.. Oncogene.

[26] Hillion J, Dhara S, Felder Sumter T, Mukherjee M, Di Cello F (2008). The HMGA1a-STAT3 axis: an “Achilles heel” for acute leukemia?. Cancer Res.

[27] Hillion J, Wood LJ, Mukherjee M, Bhattacharya R, Di Cello F (2009). Upregulation of MMP-2 by HMGA1 promotes transformation in undifferentiated, large-cell lung cancer.. Mol Cancer Res.

[28] Tesfaye A, Di Cello F, Hillion J, Ronnett B, Elbahloul R (2007). The High-Mobility Group A1 gene up-regulates cyclooxygenase 2 expression in uterine tumorigenesis..

[29] Di Cello F, Hillion J, Aderinto A, Ronnett B, Huso D (2008). COX-2 inhibitors block uterine tumorigenesis in HMGA1a transgenic mice and human uterine cancer xenografts.. Mol Cancer Ther.

[30] Fedele M, Pentimalli F, Baldassarre G, Battista S, Klein-Szanto AJ (2005). Transgenic mice overexpressing the wild-type form of the HMGA1 gene develop mixed growth hormone/prolactin cell pituitary adenomas and natural killer cell lymphomas.. Cancer Res.

[31] Shah S, Resar LMS (2011). High mobility group A1 and cancer: potential biomarker and therapeutic target.. Histol Histopathol.

[32] Schuldenfrei A, Belton A, Kowalski J, Talbot CC, Di Cello F (2011). HMGA1 drives stem cell, inflammatory pathway, and cell cycle progression genes during lymphoid tumorigenesis.. BMC Genomics.

[33] Di Cello F, Hillion J, Hristov A, Wood LJ, Mukherjee M (2008). HMGA2 participates in neoplastic transformation in human lung cancer.. Mol Cancer Res.

[34] Botchkina I, Rowehl R, Rivadeneira D, Karpeh MS, Crawford H (2009). Phenotypic subpopulations of metastatic colon cancer stem cells: genomic analysis.. Cancer Genomics and Proteomics.

[35] Erikson K, Gan C, Cheong I, Rago C, Samuels Y (2009). Genetic inactivation of *AKT1*, *AKT2*, and *PDPK1* in human colorectal cancer cells clarifies their roles in tumor growth regulation.. Proc Natl Acad Sci USA.

[36] Liau S-S, Jazag A, Whang EE (2006). HMGA1a is a determinant of cellular invasiveness & *in vitro* metastasis in pancreatic adenocarcinoma.. Cancer Res.

[37] Snippert HJ, van der Flier LG, Sato T, van Es JH, van den Born M (2010). Intestinal crypt homeostasis results from neutral competition between symmetrically dividing Lgr5 stem cells.. Cell.

[38] Kim D, Park Y, Park C, Son KC, Nam ES (1999). Expression of the HMG-I(Y) gene in human colorectal cancer.. Cancer Res.

[39] Hristov A, Delos Reyes M, Singh M, Cope L, Iacobuzio-Donahue C (2009). HMGA2 protein expression correlates with lymph node metastasis and increased tumor grade in pancreatic ductal adenocarcinoma.. Mod Pathol.

[40] Resar L, Brodsky R (2011). “Let'ing go with clonal expansion?. Blood.

[41] Ma W, Ortiz-Quintero B, Rangel R, McKeller MR, Herrera-Rodriguez S (2011). Coordinate activation of inflammatory gene networks, alveolar destruction and neonatal death in AKNA deficient mice.. Cell Res.

[42] Moliterno AR, Resar LMS (2011). AKNA: another AT-hook transcription factor “hooking-up” with inflammation.. Cell Res.

[43] Chiappetta G, Avantaggiato V, Visconti R, Fedele M, Battista S (1996). High level expression of the HMGI(Y) gene during embryonic development.. Oncogene.

