# Supplementary figures and images for: HMGA1 Induces Intestinal Polyposis in Transgenic Mice and Drives Tumor Progression and Stem Cell Properties in Colon Cancer Cells
Source: PLoS One. 2012 Jan 20;7(1):e30034. doi: 10.1371/journal.pone.0030034 (PMC3262796; doi:10.1371/journal.pone.0030034)

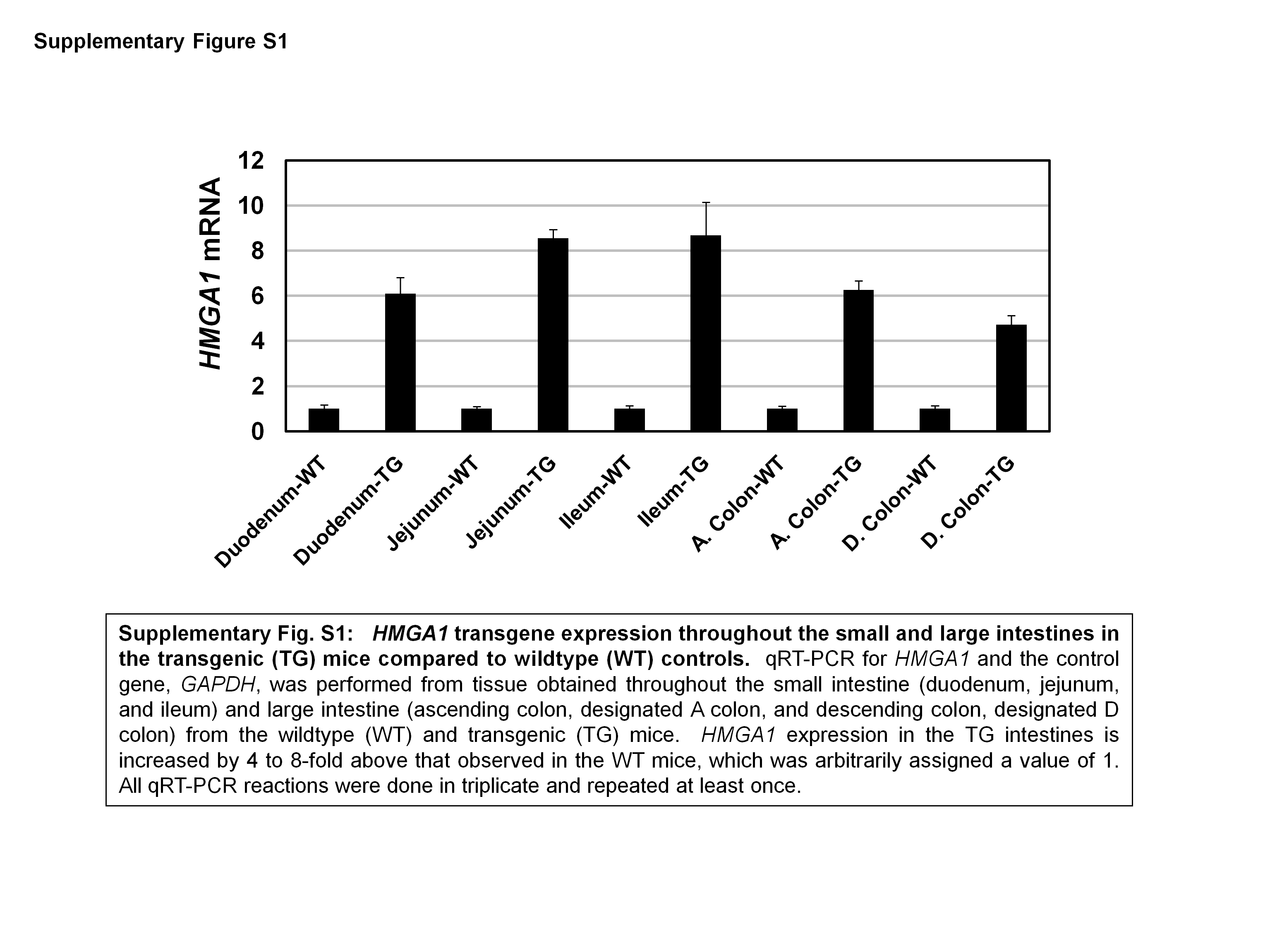

Supplement: Figure S1 — HMGA1 transgene expression throughout the small and large intestines in the transgenic (TG) mice compared to wildtype (WT) controls. qRT-PCR for HMGA1 and the control gene, GAPDH, was performed from tissue obtained throughout the small intestine (duodenum, jejunum, and ileum) and large intestine (ascending colon, designated A colon, and descending colon, designated D colon) from the TG and WT mice. HMGA1 expression in the TG intestines is increased by 4 to 8-fold above that observed in the WT mice, which was arbitrarily assigned a value of 1. All qRT-PCR reactions were done in triplicate and repeated at least once. (TIF) [file pone.0030034.s001.tif]

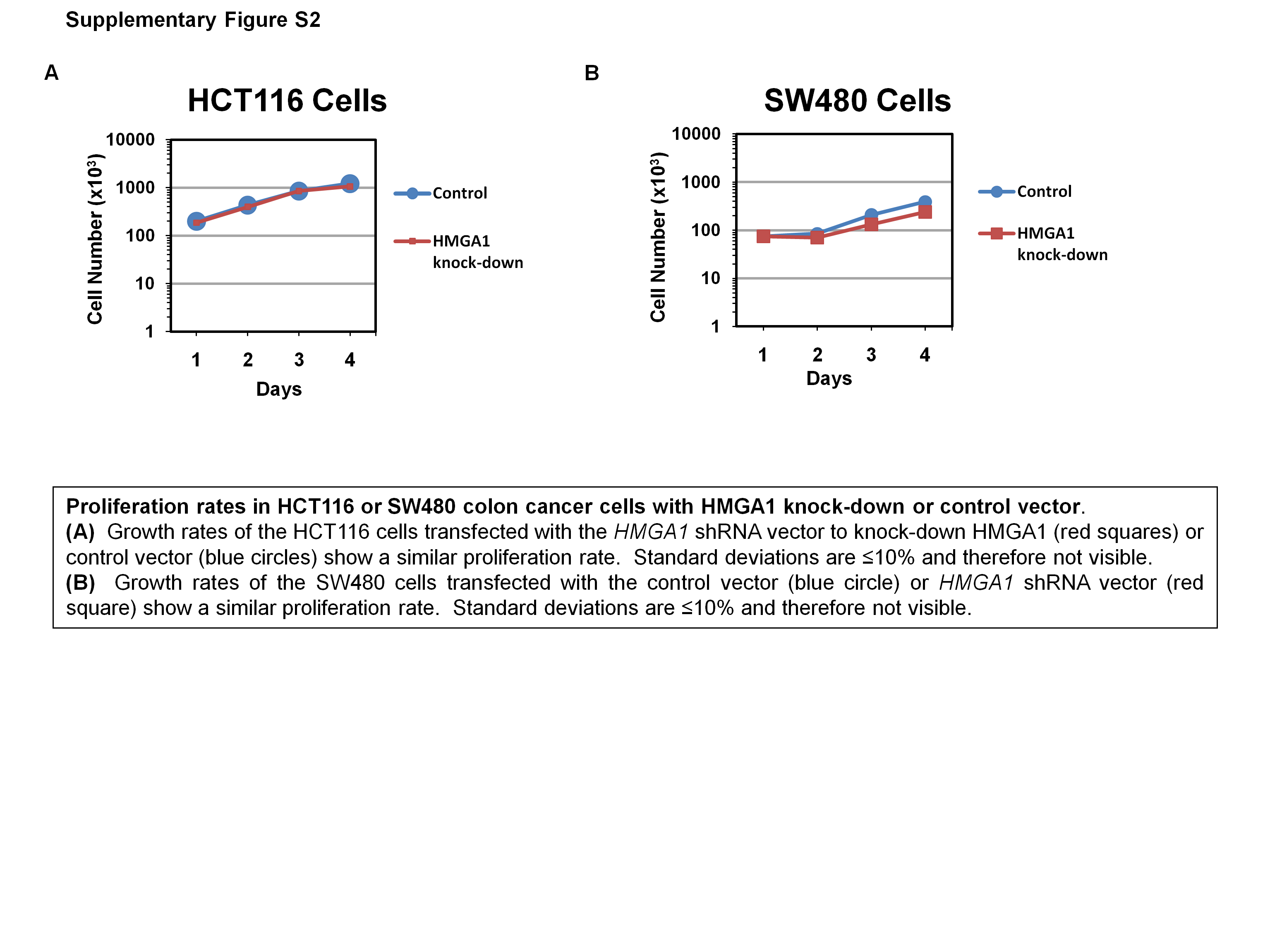

Supplement: Figure S2 — Proliferation rates in HCT116 or SW480 colon cancer cells with HMGA1 knock-down or control vector. (A) Growth rates of the HCT116 cells transfected with the HMGA1 shRNA vector to knock-down HMGA1 (red squares) or control vector (blue circles) show a similar proliferation rate. Standard deviations are ≤10% and therefore not visible. (B) Growth rates of the SW480 cells transfected with the control vector (blue circles) or HMGA1 shRNA vector (red squares) show a similar proliferation rate. Standard deviations are ≤10% and therefore not visible. (TIF) [file pone.0030034.s002.tif]
