# Supplementary material for: HMGA1 Induces Intestinal Polyposis in Transgenic Mice and Drives Tumor Progression and Stem Cell Properties in Colon Cancer Cells
Source: PLoS One. 2012 Jan 20;7(1):e30034. doi: 10.1371/journal.pone.0030034 (PMC3262796; doi:10.1371/journal.pone.0030034)

## Slide 1
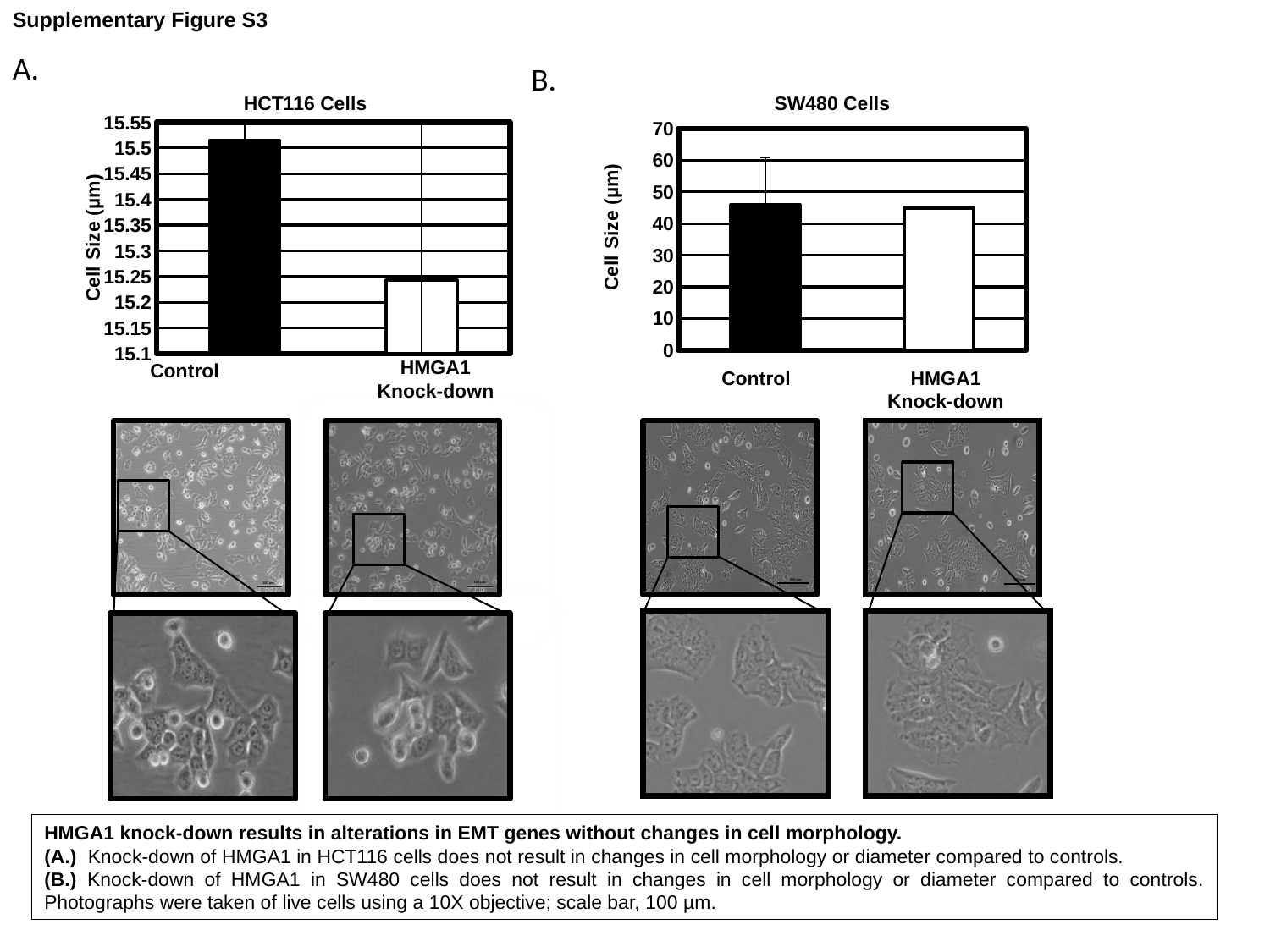

Supplementary Figure S3
A.
B.
HCT116 Cells
SW480 Cells
### Chart
| Category | |
|---|---|
### Chart
| Category | |
|---|---|Cell Size (µm)
Cell Size (µm)
HMGA1
Knock-down
Control
Control
HMGA1
Knock-down
 100 µm
 100 µm
 100 µm
 100 µm
HMGA1 knock-down results in alterations in EMT genes without changes in cell morphology.
(A.) Knock-down of HMGA1 in HCT116 cells does not result in changes in cell morphology or diameter compared to controls.
(B.) Knock-down of HMGA1 in SW480 cells does not result in changes in cell morphology or diameter compared to controls. Photographs were taken of live cells using a 10X objective; scale bar, 100 µm.

## Slide 2
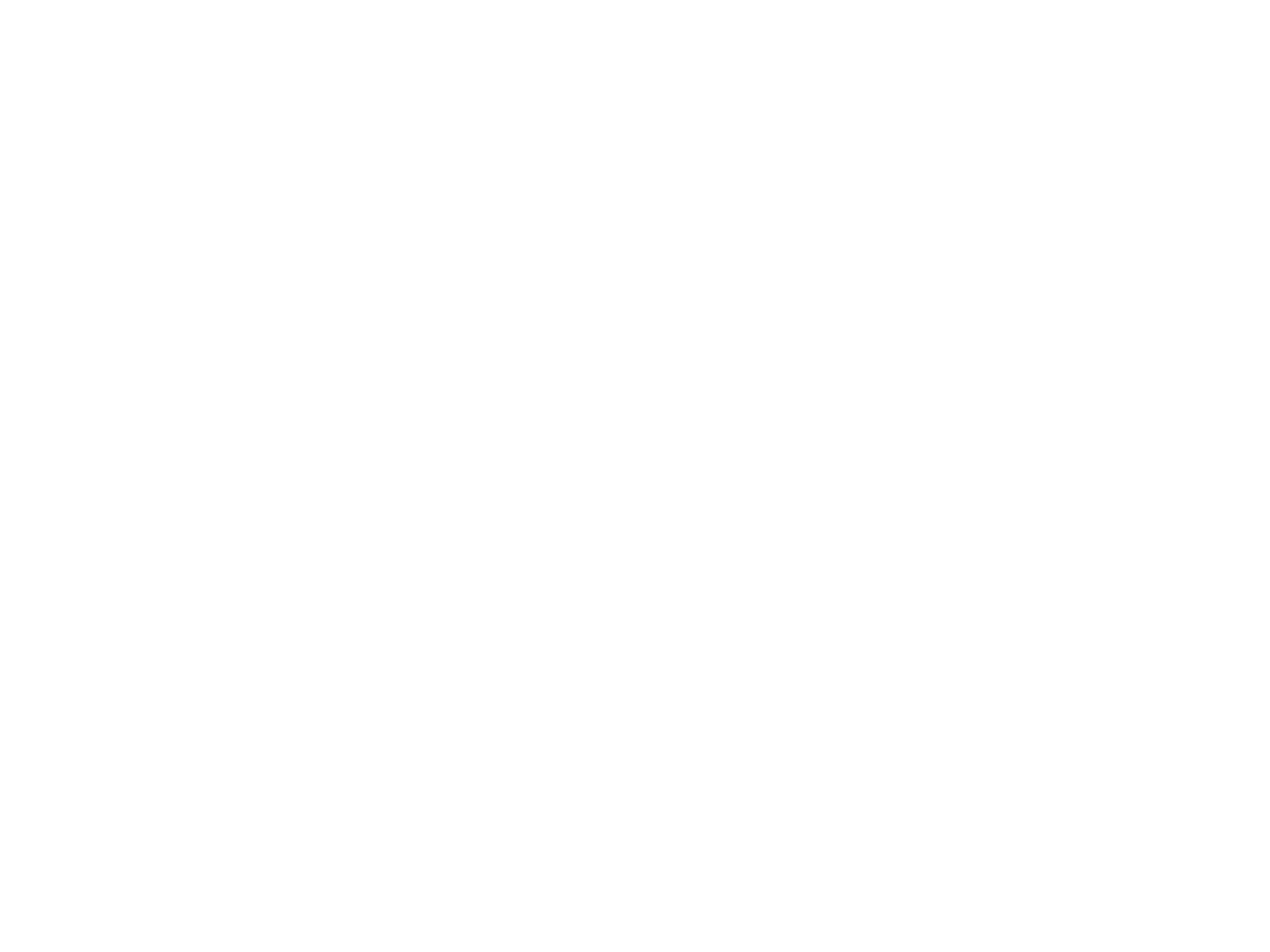

Supplement: Figure S3 — HMGA1 knock-down results in alterations in EMT genes without changes in cell morphology. (A) Knock-down of HMGA1 in HCT116 cells does not result in changes in cell morphology or diameter compared to controls. (B) Knock-down of HMGA1 in SW480 cells does not result in changes in cell morphology or diameter compared to controls. Photographs were taken of live cells using a 10× objective; scale bar, 100 µm. (PPTX) [file pone.0030034.s003.pptx]
